# Photosystem II Is More Sensitive than Photosystem I to Al^3+^ Induced Phytotoxicity

**DOI:** 10.3390/ma11091772

**Published:** 2018-09-19

**Authors:** Julietta Moustaka, Georgia Ouzounidou, Ilektra Sperdouli, Michael Moustakas

**Affiliations:** 1 Department of Botany, Aristotle University of Thessaloniki, GR-54124 Thessaloniki, Greece; ilektras@bio.auth.gr; 2 Department of Plant and Environmental Sciences, University of Copenhagen, Thorvaldsensvej 40, DK-1871 Frederiksberg C, Denmark; moustaka@plen.ku.dk; 3 Institute of Food Technology, Hellenic Agricultural Organization—Demeter, 1 S. Venizelou Str., GR-14123 Lycovrissi, Greece; geouz@yahoo.gr; 4 Institute of Plant Breeding and Genetic Resources, Hellenic Agricultural Organisation–Demeter, Thermi, GR-57001 Thessaloniki, Greece; 5 Division of Botany, Department of Biology, Faculty of Science, Istanbul University, 34134 Istanbul, Turkey

**Keywords:** aluminium, chlorophyll fluorescence, durum wheat, excitation pressure, non-photochemical quenching, photosynthesis, photoprotection, photoinhibition, reactive oxygen species, triticale

## Abstract

Aluminium (Al) the most abundant metal in the earth’s crust is toxic in acid soils (pH < 5.5) mainly in the ionic form of Al^3+^ species. The ability of crops to overcome Al toxicity varies among crop species and cultivars. Here, we report for a first time the simultaneous responses of photosystem II (PSII) and photosystem I (PSI) to Al^3+^ phytotoxicity. The responses of PSII and PSI in the durum wheat (*Triticum turgidum* L. cv. ‘Appulo E’) and the triticale (X Triticosecale Witmark cv. ‘Dada’) were evaluated by chlorophyll fluorescence quenching analysis and reflection spectroscopy respectively, under control (−Al, pH 6.5) and 148 μM Al (+Al, pH 4.5) conditions. During control growth conditions the high activity of PSII in ‘Appulo E’ led to a rather higher electron flow to PSI, which induced a higher PSI excitation pressure in ‘Appulo E’ than in ‘Dada’ that presented a lower PSII activity. However, under 148 μM Al the triticale ‘Dada’ presented a lower PSII and PSI excitation pressure than ‘Appulo E’. In conclusion, both photosystems of ‘Dada’ displayed a superior performance than ‘Appulo E’ under Al exposure, while in both cultivars PSII was more affected than PSI from Al^3+^ phytotoxicity.

## 1. Introduction

Aluminium (Al) is considered as the most abundant metal in the earth’s crust, comprising approximately 7% of the soil [1,2]. Although Al is nontoxic as a metal, with very low solubility in the neutral pH range (6.0–8.0), its solubility increases and becomes toxic to all living cells under acidic or alkaline pH conditions where is present mainly in the ionic form of Al^3+^ species (at pH < 5.5) or as aluminate Al[OH]_4_^−^ (at pH > 8.5) [3,4]. Aluminium toxicity is limiting crop production on acid soils through inhibition of root elongation, which occurs within hours of exposure to Al^3+^, disturbance of nutrient uptake and other metabolic functions, affecting also the process of photosynthesis [5,6,7,8,9,10,11,12,13,14,15,16,17,18,19,20]. Cereals differ significantly in their response to Al toxicity and genetic variation has been found between species as well as between cultivars [21,22,23], revealing distinct Al-tolerance mechanisms [2,16,18,24,25].

In the form of the trivalent cation Al^3+^, that is toxic to most plants at relatively low concentrations, it is the main limiting factor in the world’s arable non-irrigated crop production to over 40% [26,27]. With the world’s population forecasted to reach nine billion by 2050, cereal production needs to increase by 50% by 2030 [28]. Consequently, increasing cereal yields is now one of the top priorities for agricultural research [28]. Since plant production is driven by photosynthesis, studying Al^3+^ toxicity effects on photosynthesis has the potential to increase cereal yields by understanding the factors that influence negatively the molecular mechanism of absorbed light energy utilization.

Photosynthesis is the process by which organisms convert the absorbed solar energy into chemical energy via photosystem II (PSII) and photosystem I (PSI). Light reactions of photosynthesis are driven by the cooperation of PSII and PSI that work coordinately to transfer photosynthetic electrons efficiently and are located in the photosynthetic membranes of chloroplasts, the thylakoids [29,30]. Chloroplasts exhibit stacked and unstacked thylakoid membranes, designated as grana and stroma thylakoids, respectively [29,31]. The two photosystems, PSI and PSII, are laterally and functionally separated mainly in stroma (non-appressed) and grana (appressed) thylakoid membranes, respectively [29,30], that allows the regulation of the excitation energy distribution between the two photosystems [32]. Photosystem II and PSI are working in connection in the linear electron transport catalyzing the transfer of electrons from H_2_O to NADP^+^ through the formation of strong reductants, and of a proton gradient that is used to drive ATP synthesis [33,34].

Aluminium toxicity has been shown to reduce the photochemical efficiency of PSII in several plant species [35,36,37,38,39,40] by causing inhibition of electron transfer between the first stable electron acceptor of PSII, quinone A (Q_A_) and the quinone B (Q_B_) [15], and closing PSII reaction centers (RCs) [6,37,38,41,42]. Thus, Al-toxicity increases the percentage of closed PSII RCs and reduces the rate of photosynthesis with subsequent reduced growth and development [15,42,43]). However, Al-resistant cultivars keep a larger fraction of PSII RCs in an open configuration [43]. Al^3+^ concentrations resulted in a reduction of the energy transfer from light harvesting complex (LHCI) to RCs of PSI, followed by an impairment of PSI RCs and electron transfer of PSI [44].

Absorption of more light than what can be used to drive photosynthesis, causes photodamage to the photosynthetic apparatus and the light-processing structures, primarily PSII, resulting in a decrease in the photosynthetic activity causing reduced plant growth and productivity [45,46]. Among the photoprotective mechanisms that plants have developed to counteract the effects of excessive harmful energy is the dissipation of excessive energy as heat and the scavenging of reactive oxygen species (ROS) by enzymatic and non-enzymatic antioxidant molecules [47,48,49,50]. Dissipation of the excess light energy as heat in the antenna or PSII RCs is believed to be the main mechanism that plants use to deal with excessive light energy and this process is called non-photochemical quenching (NPQ) [47,51,52,53].

Chlorophyll fluorescence measurements and in particular measurements of PSII excitation pressure, that is the redox state of the plastoquinone (PQ) pool, have been proposed as a sensitive bio-indicator to measure Al effects on plants [43]. Chlorophyll fluorescence quenching analysis has been extensively applied as a probe of photosynthesis research and has been successfully used to assess the changes in the function of PSII under different environmental conditions [54,55,56]. In addition, the measurement of the fraction of closed and open reaction centers of PSI can be evaluated by reflection spectroscopy [57,58].

A number of studies as already mentioned have examined the functioning of PSII under Al toxicity [6,35,36,37,38,41,42,43], but the functioning of PSI under Al toxicity, as far as we know, was investigated only once [44]. Nevertheless, due to differences in the experimental conditions, it is problematic to acquire comprehensive information regarding the proportional resistance to Al toxicity of the two photosystems if they have not been examined concurrently. To the best of our knowledge, a simultaneous comparative study of the two photosystems to Al toxicity has not been addressed. Here, we report the concurrent responses of PSII and PSI to Al^3+^ toxicity, in the durum wheat (*Triticum turgidum* L. cv. ‘Appulo E’) and the triticale (X Triticosecale Witmark cv. ‘Dada’). Triticale is considered as more Al-tolerant species than durum wheat (*Triticum turgidum*); this difference is largely attributed to its superior ability to grow better under acidic conditions [25].

## 2. Materials and Methods

### 2.1. Plant Material and Growth Conditions

Durum wheat (*Triticum turgidum* L. cv. ‘Appulo E’) and the triticale (X Triticosecale Witmark cv. ‘Dada’) were used to compare the tolerance of the two photosystems to Al toxicity. Seeds obtained from the Institute of Plant Breeding and Genetic Resources, Thermi, Greece, germinated at 22 ± 1 °C for 2 d in the dark. The germinated seeds were transferred in a growth chamber and mounted on nylon-mesh floats on plastic vessels filled with nutrient solution at pH 6.5 [23]. The seedlings were grown in hydroponic culture at controlled environmental conditions as described previously [43].

### 2.2. Al Treatment

Al was supplied at 148 μM as KAl(SO_4_)_2_12H_2_O for 14 days. Al-containing pots (nutrient solution plus 148 μM Al) were acidified initially to pH 4.5 with 1N HCl [25], while growth solutions of control plants (nutrient solution only) were adjusted to pH 6.5. According to the GEOCHEM-EZ speciation programme [59] the free Al^3+^ activities were calculated to be 16.8 μM [43].

### 2.3. Lipid Peroxidation Measurements

The level of lipid peroxidation of controls and 14-days Al^3+^ treated plants was measured as malondialdehyde (MDA) content, as described previously [60], according to the method of Heath and Packer [61]. The concentration of MDA was calculated from the difference of the absorbance at 532 and 600 nm and expressed as nmol (MDA) g^−1^ fresh weight.

### 2.4. Measurements of Chlorophyll a Fluorescence

**Figure 1:**
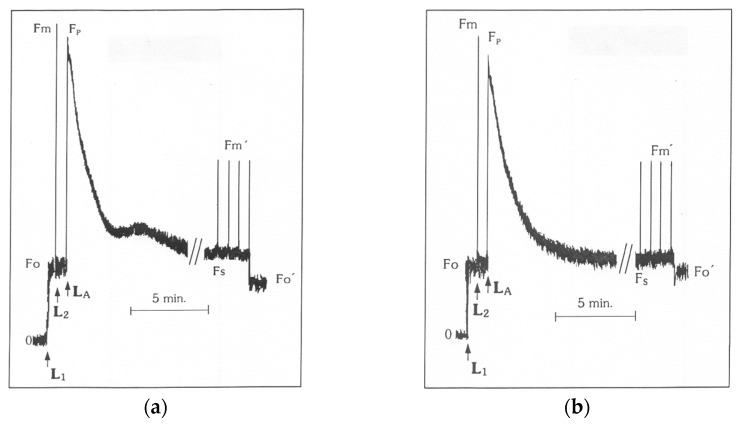
Typical modulated fluorescence signals obtained by the triticale (X Triticosecale Witmark cv. ‘Dada’) after 20 min dark adaptation; (**a**) control leaves from plants in the nutrient solution at pH 6.5; and (**b**) leaves from plants in the nutrient solution plus 148 μM Al at pH 4.5; L_1_, arrow denotes onset of a weak modulated light beam; L_2_, arrow denotes onset of a saturating light pulse (approximately 8000 μmol photons m^−2^ s^−1^); L_A_, arrow denotes continuous actinic light.

Chlorophyll a fluorescence was measured in dark-adapted (20 min) leaf samples, using a pulse amplitude modulation fluorometer (PAM, Walz, Effeltrich, Germany), as described before [35,43]. First, minimal chlorophyll *a* fluorescence (*F*_o_) was measured by application of a weak modulated light beam (L_1_) followed by a saturating light pulse (L_2_) to measure maximal chlorophyll *a* fluorescence (*F*_m_) in the dark adapted (20 min) samples. Then, by application of the actinic light (L_A_) and saturating light pulses, maximum chlorophyll *a* fluorescence in the light (F_m_′) was measured, while to assess steady-state photosynthesis (*F*_s_) values, the actinic light (L_A_) alone was applied. Minimum chlorophyll *a* fluorescence in the light (*F*_o_′) was measured immediately after turning off the actinic light (L_A_) (Figure 1). The calculated chlorophyll fluorescence parameters with their definitions are given in Table 1.

### 2.5. Measurements of Leaf Absorbance Changes at 820 nm

A Hansatech P_700_+ measuring system was employed to monitor light-induced changes in leaf absorbance at around 820 nm according to Havaux et al. [57], as described before [58]. The fraction of closed PSI reaction centers (B_1_) was calculated as: B_1_ = ΔS/(ΔS)max = (Rfr − R’)/(Rfr − R).

### 2.6. Statistical Analysis

Data are presented as the mean ± SD. Statistical analysis was performed using the Student’s *t*-test. Differences were considered statistically significant at *p* < 0.05.

## 3. Results

### 3.1. Allocation of the Absorbed Light Energy in PSII under Normal Growth and Al^3+^ Exposure

**Figure 2:**
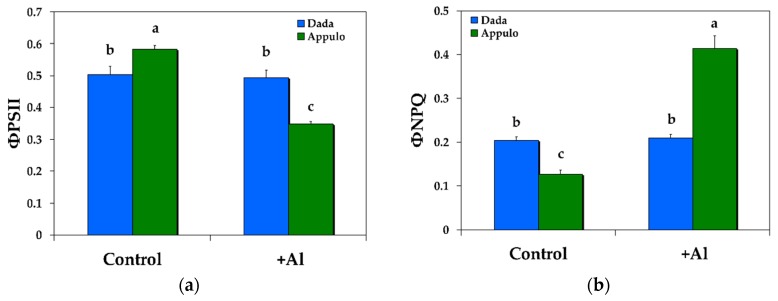
Changes in the balance between light capture and energy use in the triticale ‘Dada’ and the durum wheat ‘Appulo E’; (**a**) the quantum efficiency of photosystem II (PSII) photochemistry (photochemical utilization) (Φ*_PSΙΙ_*); and (**b**) the quantum yield for dissipation by down regulation in PSII (regulated heat dissipation, a loss process serving for protection) (Φ*_NPQ_*); under normal growth conditions (control) and under Al^3+^ exposure (+Al). Error bars on columns are standard deviations based on five leaves from five plants. Columns with different letters are statistically different (*p* < 0.05).

**Figure 3:**
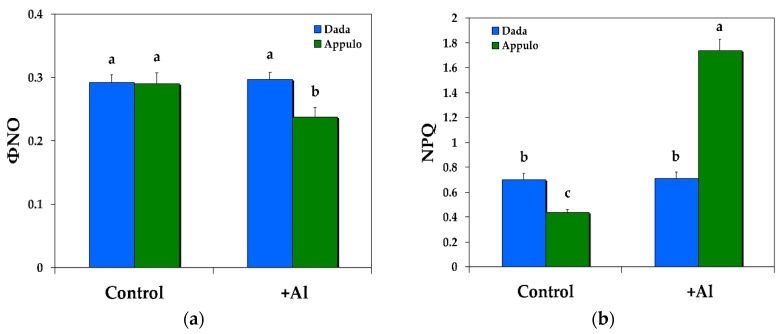
Changes in the quantum yield of non-regulated energy dissipated in PSII (non-regulated heat dissipation, a loss process due to PSII inactivity) (Φ*_NO_*) (**a**); and changes in non-photochemical fluorescence quenching (NPQ) (**b**); in the triticale ‘Dada’ and the durum wheat ‘Appulo E’, under normal growth conditions (control) and under Al^3+^ exposure (+Al). Error bars on columns are standard deviations based on five leaves from five plants. Columns with different letters are statistically different (*p* < 0.05).

Under control growth conditions at pH 6.5, the durum wheat ‘Appulo E’ presented higher effective quantum yield of photochemical energy conversion in PSII (Φ*_PSΙΙ_*) (Figure 2a) and lower quantum yield of regulated non-photochemical energy loss in PSII (Φ*_NPQ_*) (Figure 2b), with no difference in the quantum yield of non-regulated energy loss in PSII (Φ*_NO_*) (Figure 3a), compared with triticale ‘Dada’. Under 148 μM Al at pH 4.5 the triticale ‘Dada’ had higher Φ*_PSΙΙ_* and lower Φ*_NPQ_* (Figure 2), but higher Φ*_NO_* (that did not differ from control conditions) (Figure 3a), than the durum wheat ‘Appulo E’. However, ‘Appulo E’ due to the efficient photoprotective mechanism, that is the quantum yield for dissipation by down regulation in PSII, possessed lower Φ*_NO_* even though from control conditions (Figure 3a).

### 3.2. Non-Photochemical Quenching under Normal Growth and Al^3+^ Exposure

The triticale ‘Dada’ had higher non-photochemical fluorescence quenching (NPQ) under control growth conditions (pH 6.5) than the durum wheat ‘Appulo E’ but under 148 μM Al at pH 4.5 it was the reverse (Figure 3b).

### 3.3. Electron Transport Rate and the Redox State of PSII under Normal Growth and Al^3+^ Exposure

**Figure 4:**
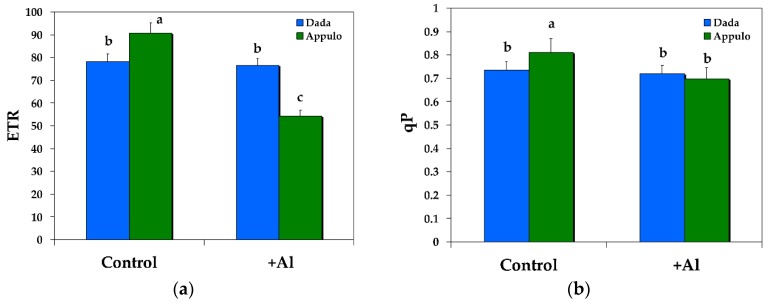
Changes in the relative PSII electron transport rate (ETR) (**a**); and changes in the photochemical fluorescence quenching, that is the relative reduction state of Q*_A_*, reflecting the fraction of open PSII reaction centers (*q*_p_) (**b**); in the triticale ‘Dada’ and the durum wheat ‘Appulo E’, under normal growth conditions (control) and under Al^3+^ exposure (+Al). Error bars on columns are standard deviations based on five leaves from five plants. Columns with different letters are statistically different (*p* < 0.05).

Under control growth conditions the durum wheat ‘Appulo E’ presented higher electron transport rate (ETR) (Figure 4a) and a more oxidized redox state of PSII (*q*_p_) (Figure 4b), than the triticale ‘Dada’. Under Al exposure the triticale ‘Dada’ had higher ETR than the durum wheat ‘Appulo E’ (Figure 4a), but the same redox state of PSII (*q*_p_) (Figure 4b) with durum wheat ‘Appulo E’.

### 3.4. The Maximum PSII Quantum Efficiency (F_v_/F_m_) and PSII Maximum Efficiency in Light (F_v_’/F_m_’) under Normal Growth and Al^3+^ Exposure

**Figure 5:**
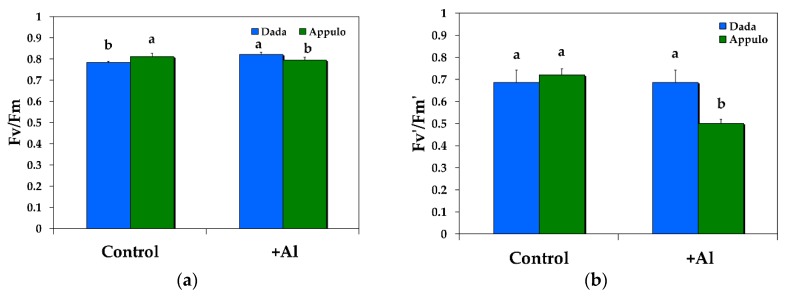
The maximum quantum efficiency of PSII (*F*_v_/*F*_m_) (**a**); and the maximum efficiency of PSII in the light (*F*_v_’/*F*_m_’) (**b**); in the triticale ‘Dada’ and the durum wheat ‘Appulo E’, under normal growth conditions (control) and under Al^3+^ exposure (+Al). Error bars on columns are standard deviations based on five leaves from five plants. Columns with different letters are statistically different (*p* < 0.05).

The maximum quantum efficiency of PSII (*F*_v_/*F*_m_) under normal growth conditions was higher in the durum wheat ‘Appulo E’, but under 148 μM Al it was higher in the triticale ‘Dada’ (Figure 5a). The maximum efficiency of PSII in the light (*F*_v_’/*F*_m_’) was similar under control growth conditions (Figure 5b), but under 148 μM Al it was higher in the triticale ‘Dada’ suggesting a higher quantum yield of the open, functional reaction centers, than in the durum wheat ‘Appulo E’ (Figure 5b).

### 3.5. Oxidative Damage under Normal Growth and Al^3+^ Exposure

**Figure 6:**
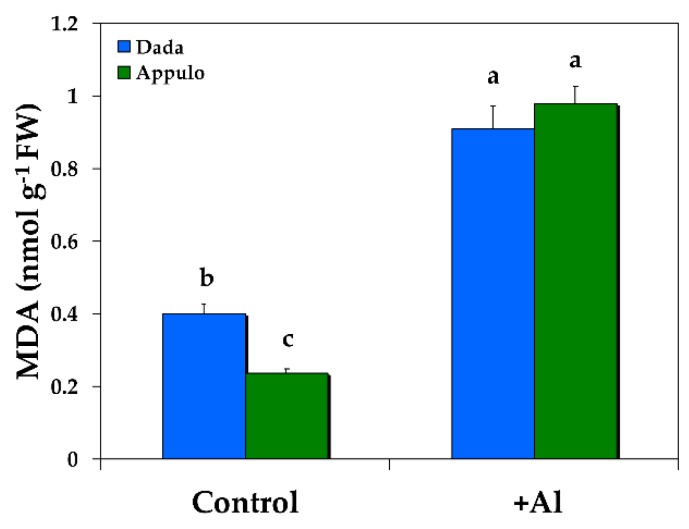
Changes in the level of lipid peroxidation measured as malondialdehyde (MDA) content and expressed as nmol (MDA) g^−1^ fresh weight in the triticale ‘Dada’ and the durum wheat ‘Appulo E’, under normal growth conditions (control) and under Al^3+^ exposure (+Al). Error bars on columns are standard deviations based on five leaves from five plants. Columns with different letters are statistically different (*p* < 0.05).

Under Al exposure the level of lipid peroxidation measured as malondialdehyde (MDA) content and expressed as nmol (MDA) g^−1^ fresh weight increased compared with control growth conditions, but it was the same in both the triticale ‘Dada’ and the durum wheat ‘Appulo E’, while under normal growth conditions it was higher in ‘Dada’ (Figure 6).

### 3.6. Excitation Pressure in PSI and PSII under Normal Growth and Al^3+^ Exposure

The fraction of closed PSI reaction centers (B_1_) or PSI excitation pressure under both control growth conditions and Al exposure was higher in ‘Appulo E’ (Table 2), while the fraction of closed PSII reaction centers (PSII excitation pressure) under control growth conditions was higher in the triticale ‘Dada’, but under Al exposure was higher in ‘Appulo E’ (Table 2). Under 148 μM Al, PSII excitation pressure in both triticale ‘Dada’ and durum wheat ‘Appulo E’ was higher than PSI excitation pressure (Table 2).

## 4. Discussion

In a hydroponic solution as summarized by Famoso et al. [2], Al may be found either (a) as free Al^3+^, that actively inhibits root growth; (b) precipitated with other elements and essentially non-toxic to plant growth; (c) different hydroxyl Al monomers also non-toxic to roots [62]; or (d) complexed with other elements in an equilibrium between its active and inactive states. Thus, the degree of Al toxicity to plants is primarily related to the activity of free Al^3+^ in solution [63]. In our experiment, according to the GEOCHEM-EZ speciation program [59], the free Al^3+^ activities in the nutrient solutions were calculated to be 16.8 μM.

The significant lower quantum efficiency of PSII photochemistry in ‘Dada’ (Φ*_PSII_*) under control growth conditions (Figure 2a) was compensated by a significant higher regulated heat dissipation, a loss process serving for protection (Φ*_NPQ_*) (Figure 2b), that was sufficient enough to retain the same quantum yield of non-regulated energy dissipated in PSII (Φ*_NO_*) in both ‘Dada’ and ‘Appulo E’ (Figure 3a). Under Al exposure we observed a reverse situation, with the significant higher photoprotective heat dissipation (Φ*_NPQ_*) in ‘Appulo E’ (Figure 2b) not only to compensate the significant lower quantum efficiency of PSII photochemistry (Φ*_PSII_*) (Figure 2a), but even more, to lower the quantum yield of non-regulated energy dissipated in PSII (Φ*_NO_*) compared to ‘Dada’ (Figure 3a).

The most vulnerable component of the photosynthetic machinery to abiotic stresses is considered to be PSII [64]. However, despite the fact that PSI was shown to be more resistant to mild water deficit than PSII, it was heavily damaged by prolonged water deficit [64]. PSI is impaired when electron flow from PSII to PSI exceeds the capability of PSI electron carriers to manage the electrons [65,66]. Proton gradient (ΔpH)-dependent slow-down of electron transfer from PSII to PSI protects PSI from excess electrons [66]. As occurred in our experiment, PSI in Appulo E was more inhibited under control growth conditions than in Dada. During control growth conditions the high activity of PSII in Appulo E led to a rather higher electron flow to PSI, causing probably the formation of ROS within PSI complex [67,68], which induced a higher PSI excitation pressure in Appulo E than in Dada (Table 2) that presented a lower PSII photochemistry (Figure 2a) and lower PSI excitation pressure (Table 2). This higher PSI photoinhibition in Appulo E than in Dada under control growth conditions was alleviated by the absence of PSII photoinhibition in Appulo E as indicated by the *F*_v_/*F*_m_ value (Figure 5). The absence of the photoprotective mechanism of NPQ in Appulo E under control growth conditions (Figure 3b), that slows-down the electron transfer from PSII to PSI, could not protect PSI from excess electrons. However, this absence of the photoprotective mechanism of NPQ in Appulo E (Figure 3b) did not cause any problem on the fraction of open PSII reaction centers (Figure 4b). Thus, under control growth conditions Appulo E shows lower PSII excitation pressure and a better PSII function, despite a higher PSI photoinhibition. Hence, regardless of the ROS formation within PSI complex in Appulo E, the level of lipid peroxidation, measured by MDA accumulation that reflects ROS formation and corresponds to oxidative damage, was shown to be less than in Dada, under control growth conditions (Figure 6). Current evidence suggests that ROS production can serve as the signal that triggers the expression of genes that may serve to alleviate electron pressure on the reducing side of PSI [69].

After Al exposure, the electron flow from PSII to PSI in ‘Appulo E’ was suppressed, but excitation pressure was increased in both photosystems, although more in PSII. This slow-down of electron transfer from PSII to PSI in ‘Appulo E’ protects PSI from excess electrons. Α proper regulation of ETR is crucial in the protection of PSI against photoinhibition [70]. However, PSI photoinhibition may represent a kind of protective mechanism against over-reduction of PSI acceptor side, diminishing creation of huge amount of ROS and avoiding extensive cell injury [71,72]. The controlled photoinhibition of PSII in ‘Appulo E’, under Al exposure, as indicated by the *F*_v_/*F*_m_ value (Figure 5a), was also able to protect PSI from permanent photodamage [66].

The high excitation pressure in PSII (1 − qp) under Al exposure, observed in ‘Appulo E’ (Table 2), indicates an imbalance between energy supply and demand [73]. However, the significant increase of NPQ processes in PSII (Figure 3b), that reflects the dissipation of excess excitation energy in the form of harmless heat [47,51,52,74], seems that protected ‘Appulo E’ plants under Al exposure from the destructive effects of ROS. It appears that under Al exposure, NPQ increase in PSII was sufficient enough to protect ‘Appulo E’ plants from ROS production since the quantum yield of non-regulated non-photochemical energy loss (Φ*_NO_*) decreased significantly (Figure 3a), thus exhibited lower singlet oxygen (^1^O_2_) production. The quantum yield of non-regulated non-photochemical energy loss (Φ*_NO_*) consists of chlorophyll fluorescence internal conversions and intersystem crossing, which leads to the formation of ^1^O_2_ via the triplet state of chlorophyll (^3^chl*) [75,76,77]. The increased NPQ in ‘Appulo E’ under Al exposure (Figure 3b) was also capable to keep the same fraction of open reaction centers as in Dada (Figure 4b) and also the same level of lipid peroxidation (Figure 6), thus the same degree of oxidative damage. The photoprotective mechanism of NPQ can divert absorbed light to other processes, such as thermal dissipation, preventing the photosynthetic apparatus from oxidative damage [47,48,78,79,80,81].

## 5. Conclusions

In conclusion, we confirmed that the triticale cv. ‘Dada’ was more tolerant to Al phytotoxicity than durum wheat ‘Appulo E’, as reflected by the better PSII functionality under Al acidic conditions. However, under normal growth conditions (−Al, pH 6.5), durum wheat ‘Appulo E’ displayed a better PSII functionality. Yet, under Al exposure, PSII was more affected than PSI from Al^3+^ phytotoxicity in both cultivars.

## Figures and Tables

**Table 1:** Definitions of the calculated chlorophyll fluorescence parameters with their calculation formula.

Chlorophyll Fluorescence Parameter	Definition	Calculation
*F*_v_/*F*_m_	The maximum quantum efficiency of photosystem II (PSII) photochemistry	Calculated as (*F*_m_ − *F*_o_)/*F*_m_
*F*_v_’/*F*_m_’	The PSII maximum efficiency is an estimate of the maximum efficiency of PSII photochemistry at a given PPFD (photosynthetic photon flux density)	Calculated as (*F*_m_’ − *F*_o_’)/*F*_m_’
Φ*_PSII_*	The effective quantum yield of photochemical energy conversion in PSII estimating the efficiency at which light absorbed by PSII is used for photochemistry, that means is used for reduction of the primary acceptor of PSII quinone A (QA)	Calculated as (*F*_m_’ − *F*_s_)/*F*_m_’
*q*_p_	The photochemical quenching is a measure of the fraction of open PSII reaction centers, that is the redox state of QA	Calculated as (*F*_m_’ − *F*_s_)/(*F*_m_’ − *F*_o_’)
NPQ	The non-photochemical quenching that reflects heat dissipation of excitation energy	Calculated as (*F*_m_ − *F*_m_’)/*F*_m_’
ETR	The relative PSII electron transport rate	Calculated as Φ*_PSII_* × PPFD × c × abs, where c is 0.5 since the absorbed light energy is assumed to be equally distributed between PSII and PSI, and abs is the total light absorption of the leaf taken as 0.84.
Φ*_NPQ_*	The quantum yield of regulated non-photochemical energy loss in PSII, that is the quantum yield for dissipation by down regulation in PSII	Calculated as *F*_s_/*F*_m_’ − *F*_s_/*F*_m_
Φ*_NO_*	The quantum yield of non-regulated energy loss in PSII, a loss process due to PSII inactivity	Calculated as *F*_s_/*F*_m_
1 − *q*_p_	Excitation pressure of PSII, or the fraction of closed PSII reaction centers	Calculated as 1 − *q*_p_

**Table 2:** PSI and PSII excitation pressure in the triticale ‘Dada’ and the durum wheat ‘Appulo E’, under normal growth conditions, under Al^3+^ exposure, and the percentage change.

Chlorophyll Fluorescence Parameter	Control Growth	+148 μM Al	Change %
B1 (excitation pressure in PSI) ‘Dada’	0.259	0.265	+2.3
B1 (excitation pressure in PSI) ‘Appulo E’	0.280	0.339	+21.0
1-qp (excitation pressure in PSII) ‘Dada’	0.266	0.281	+5.6
1-qp (excitation pressure in PSII) ‘Appulo E’	0.188	0.303	+61.2
